# Potential systematic uncertainties in IGRT when FBCT reference images are used for pancreatic tumors

**DOI:** 10.1120/jacmp.v16i3.5257

**Published:** 2015-05-08

**Authors:** Ahmad Amoush, May Abdel‐Wahab, Mohamed Abazeed, Ping Xia

**Affiliations:** ^1^ Department of Radiation Oncology Cleveland Clinic Cleveland OH USA

**Keywords:** IGRT, pancreas, AIP, FBCT, CBCT

## Abstract

The purpose of this study was to quantify the systematic uncertainties resulting from using free breathing computed tomography (FBCT) as a reference image for image‐guided radiation therapy (IGRT) for patients with pancreatic tumors, and to quantify the associated dosimetric impact that resulted from using FBCT as reference for IGRT. Fifteen patients with implanted fiducial markers were selected for this study. For each patient, a FBCT and an average intensity projection computed tomography (AIP) created from four‐dimensional computed tomography (4D CT) were acquired at the simulation. The treatment plan was created based on the FBCT. Seventy‐five weekly kilovoltage (kV) cone‐beam computed tomography (CBCT) images (five for each patient) were selected for this study. Bony alignment without rotation correction was performed 1) between the FBCT and CBCT, 2) between the AIP and CBCT, and 3) between the AIP and FBCT. The contours of the fiducials from the FBCT and AIP were transferred to the corresponding CBCT and were compared. Among the 75 CBCTs, 20 that had >3 mm differences in centers of mass (COMs) in any directions between the FBCT and AIP were chosen for further dosimetric analysis. These COM discrepancies were converted into isocenter shifts in the corresponding planning FBCT, and dose was recalculated and compared to the initial FBCT plans. For the 75 CBCTs studied, the mean absolute differences in the COMs of the fiducial markers between the FBCT and CBCTs were 3.3 mm±2.5 mm,3.5 mm±2.4 mm, and 5.8 mm±4.4 mm in the right–left (RL), anterior–posterior (AP), and superior–inferior (SI) directions, respectively. Between the AIP and CBCTs, the mean absolute differences were 3.2 mm±2.2 mm,3.3 mm±2.3 mm, and 6.3 mm±5.4 mm. The absolute mean discrepancies in these COMs shifts between FBCT/CBCT and AIP/CBCT were 1.1 mm±0.8 mm,1.3 mm±0.9 mm, and 3.3 mm±2.6 mm in RL, AP, and SI, respectively. This represented a potential systematic error. For the 20 CBCTs that had COM discrepancies >3 mm in any direction, the average reduction in planning target volume (PTV) coverage (PTV volume receiving 100% of prescription dose) was 5.3%±3.1% (range: 0.7%–12.8%). Using FBCT as a reference for IGRT may introduce potential interfractional systematic COM shifts if the FBCT is acquired at a different breathing phase than the average breathing phase. The potential systematic error could be significant in the SI direction and varied among patients for the other directions. AIP is a better choice of reference image set for IGRT in order to correct interfractional variations due to respiratory motion and nonrespiratory organ displacement.

PACS numbers: 87.55.D, 87.55.dk, 87.55.km

## I. INTRODUCTION

Pancreatic cancer is the fourth leading cause of cancer death in the United States, with a five‐year survival rate of just 6%.^(1)^ Radiotherapy of pancreatic cancer often applies a large planning margin in order to account for tumor motion and displacement. Several previous studies have reported the magnitude of pancreatic tumor motions using different motion management techniques, such as deep inspiration and deep expiration, cine magnetic resonance imaging (MRI), and four‐dimensional computed tomography (4D CT) imaging.^2^, ^3^, ^4^, ^5^ All of these research studies concluded that the pancreatic motion is highly variable between patients, and individualized margins should be considered for the planning target volume (PTV). Applying a large planning margin increases the treatment toxicities of nearby critical organs, primarily the duodenum, small and large bowels, stomach, kidneys, and liver.

Several studies employed various image‐guided radiation therapy (IGRT) techniques in order to assess pancreatic interfractional tumor motion with implanted markers after bony alignment. From these studies, the reference image sets for the IGRT alignments were either free breathing computed tomography (FBCT) or average intensity projection (AIP). Using FBCT as a reference, Solla et al.^(6)^ assessed the interfractional motion for 10 pancreatic patients using weekly megavoltage (MV) cone‐beam computed tomography (CBCT) images and the position of a single fiducial marker. Whitfield et al.^(7)^ used kilovoltage (kV) CBCTs in order to calculate the interfractional fiducial motions after bony alignment for 13 patients. Jayachandran et al.^(8)^ used seeds and orthogonal kV planar imaging for five patients in order to quantify the interfractional variations in pancreatic tumor motion. Using a mix of FBCTs and AIP (three patients with FBCT and 10 patients with AIP) as the reference images, Van der Horst et al.^(5)^ studied the interfractional pancreatic motion based on the implanted markers on daily kV CBCTs. All of these research studies used FBCT as the reference computed tomography (CT); however, sample size and techniques varied. It is known that FBCT is often acquired in a short time, which could capture the tumor position in any phase of the respiratory cycle. The purpose of this study is to determine the potential systematic uncertainties involved when FBCT is used as a reference image set for IGRT for patients with pancreatic tumors; the research will also quantify the associated dosimetric impact.

## II. MATERIALS AND METHODS

A total of 15 patients who underwent chemoradiotherapy for preoperative pancreatic adenocarcinoma at our institution, from 2012 to 2103, were retrospectively selected for this study. Each patient underwent fiducial marker implantation under endoscopic ultrasound guidance at least five days before the day of simulation to allow for marker settlement. Three to five markers were implanted for each patient. On the day of simulation, patients were in a supine position with arms above the head and immobilized with a blue cushion set and neck pad (Orfit Industries, Wijnegem, Belgium). Oral and intravenous (IV) contrasts were administered during CT simulation. Both FBCT and 4D CT with 10 respiratory phases were acquired using 3 mm slices on a Brilliance CT Big Bore (Philips Medical System, Eindhoven, The Netherlands).

After simulation, the gross tumor volume (GTV) was contoured on the FBCT and a margin accounting for microscopic disease was added to create the clinical target volume (CTV). A planning margin of 1 cm in all directions was further added to the CTV to create the PTV. The FBCT and 4D CT were fused using MIMvista Software (MIMvista Corp, Cleveland, OH) to ensure that the motion envelope of the tumor observed from 4D CT was inside the PTV. If it was not inside the PTV, the PTV was enlarged to cover the tumor motion envelope. All organs at risk were contoured on the FBCT, including the right and left kidneys, duodenum, small bowel, stomach, liver, lungs, and spinal cord. Typical five‐beam intensity‐modulated radiation therapy (IMRT) treatment plans were created on the FBCT for a prescription of 50.4 Gy at 1.8 Gy/fraction using the Pinnacle 9.0 treatment planning system (Philips Radiation Oncology Systems, Fitchburg, WI).

After positioning the patient on the treatment table by aligning the skin marks with the laser, a daily kV CBCT was obtained using the Elekta X‐ray volume imaging (XVI) on a Synergy system (Elekta Oncology Systems, Crawley, UK). Daily CBCT was aligned to the bony anatomy of the planning FBCT to correct for setup errors.

**Figure 1:**
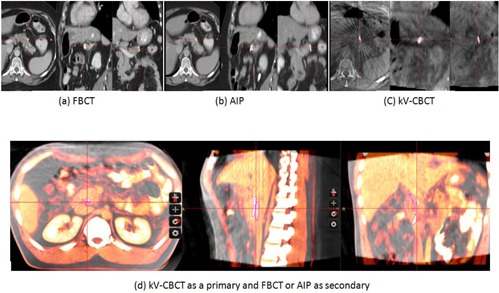
Fiducial markers shown on (a) FBCT, (b) AIP, and (c) CBCT, and (d) bony alignment without rotation correction between CBCT as the primary CT and FBCT or AIP as the secondary CT.

After treatment, five weekly CBCTs, representing the 1st, 7th, 13th, 19th, and 25th treatment fractions, were exported to the MIM workstation. The fiducial markers on the FBCT, AIP, and CBCT were manually contoured on the MIM workstation (Figs. 1(a) to 1(c)). Bony registration, without rotation corrections, using the rigid fusion algorithm and box‐based assisted alignment from the MIM software were performed 1) between the FBCT and CBCT and 2) between the AIP and CBCT (Fig. 1(d)). In order to register both FBCT and AIP to the same reference frame, we chose CBCT as the primary image set. After registration, the fiducial marker contours were transferred from the FBCT and AIP into the CBCT reference frame. The center of mass (COM) coordinates of the fiducial maker contours from FBCT, AIP and CBCT were recorded and compared.

To study the dosimetric impact of using FBCT versus AIP as a reference image set for IGRT, the discrepancies in COM coordinates between FBCT/CBCT and AIP/CBCT were translated into isocenter shifts in the corresponding treatment plan, and the dose was recalculated. Only fractions with differences >3 mm in any directions were included in dosimetric analysis as no dosimetric impact was noticed for fractions with differences <3 mm. The volume of the PTV receiving at least 100% of the prescription dose (${\text{PTV}}_{100\%}$) was analyzed and normal tissue tolerances from Quantitative Analysis of Normal Tissue Effects in the Clinic (QUANTEC) were adopted in this study as the following: 1) mean dose to liver ≤30 Gy; 2) mean dose to bilateral kidneys ≤18 Gy and volume receiving 20 Gy less than $32\%(V_{20}\leq 32\%);3)$ maximum dose to stomach <45 Gy; 4) volume of the small bowel receiving 50–54 Gy $\leq 2\%(V_{50-54}\leq 2\%)$; 5) duodenum maximum dose <60 Gy; 6) maximum spinal cord dose <45 Gy.

## III. RESULTS

**Figure 2:**
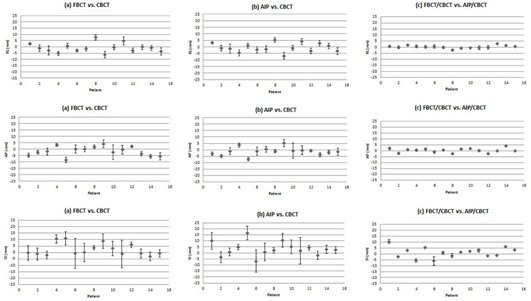
Differences in COM coordinates between (a) FBCT vs. CBCT; (b) AIP vs. CBCT, and (c) FBCT/CBCT vs. AIP/CBCT. Vertical error bars represent the standard deviations.

After alignment to the bony anatomy, the mean absolute differences in COMs of the fiducial markers between FBCT and CBCT were 3.3 mm±2.5 mm (ranging from −6.1 mm to 7.6 mm), 3.5 mm±2.4 mm (ranging from −8.4 mm to 4.2 mm), and 5.8 mm±4.4 mm (ranging from −3.2 mm to 11 mm) in the right–left (RL), anterior–posterior (AP), and superior–inferior (SI) directions, respectively. Accordingly, the mean absolute differences between AIP and CBCT were 3.2 mm±2.2 mm (ranged from −6.8 mm to 5.1 mm), 3.3 mm±2.3 mm (ranged from −7.3 mm to 5.2 mm), and 6.3 mm±5.4 mm (ranged from −6.9 mm to 16.5 mm). The detail distributions of this data are shown in Figs. 2(a) and 2(b).

The absolute mean discrepancies of the COM shifts between FBCT/CBCT versus AIP/CBCT were 1.1 mm±0.8 mm,1.3 mm±0.9 mm, and 3.3 mm±2.6 mm in the RL, AP, and SI directions, respectively. The spread in these COM shifts ranged from −2.5 mm to 2.8 mm, −2.9 mm to 3.7 mm, and −6.0 mm to 10.5 mm in the RL, AP, and SI directions, respectively (Fig. 2(c)).

Table 1 shows the dose‐volume metrics for selected normal structures from the original plans and the plans with shifted isocenters. The shifted isocenters represented potential systematic uncertainties if the FBCT was selected as a reference image for IGRT. The markers on the FBCT can be captured at a different breathing phase from what is captured during CBCT acquisition, since CBCT is always an average acquisition. All normal tissue doses from both plans satisfied the dose tolerances from QUANTIC. Significant statistical differences in the spinal cord and stomach maximum doses were noticed, but had no clinical impact. The ${\text{PTV}}_{100\%}$ and doses to normal structures depend on the location of the PTV and the magnitude and direction of the shifted isocenter.

**Table 1:** Dose‐volume metrics for normal structures for the cases that showed differences >3 mm when using different reference image sets for IGRT. The original plan was shifted by the differences between FBCT/CBCT and AIP/CBCT to create the shifted plan.

Average±SD	*Bilateral Kidneys Mean Dose (Gy)*	*Bilateral Kidneys* $\left(V_{20}\pm 32\%\right)$	*Liver Mean Dose (Gy)*	*Stomach Maximum Dose (Gy)*	*Duodenum Maximum Dose (Gy)*	*Small Bowel* $\left(V_{50-54}\leq 2\%\right)$	*Cord Maximum Dose (Gy)*
Original plan	9.1±3.9	8.5±7.3%	9.6±4.6	10.7±8.6	52.3±0.7	0	29.4±8.7
Shift plan on FBCT	9.4±4.5	9.7±8.5%	8.6±5.0	12.3±9.9	52.4±0.7	0	28.9±8.5
p‐value	0.96	0.82	0.80	0.01	0.55	n/a	0.01

**Figure 3:**
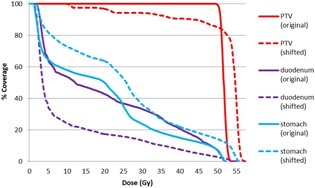
Changes in the PTV coverage and normal structure sparing for the case showing the largest reduction in ${\text{PTV}}_{100\%}$ (Patient # 1/fraction # 1 in Table 2) between the original plan (solid curves) and the shifted plan (dashed curves).

In 20 of 75 fractions, the COM discrepancies in the frame of CBCT between FBCT and AIP showed a difference >3 mm in any direction. This difference represents systematic uncertainty in the treatment isocenter if the daily CBCT is aligned to the markers of the FBCT. After applying these isocenter shifts in the corresponding treatment plans on the FBCT, the average ${\text{PTV}}_{100\%}$ was reduced by 5.3%±3.1% (range: 0.7%–12.8%) in these 20 fractions. Table 2 lists 6 fractions (out of 20) with the largest reduction in ${\text{PTV}}_{100\%}$. Figure 3 shows the dose‐volume histogram (DVH) for Patient #1, fraction #1 from Table 2.

**Table 2:** Change in PTV coverage using FBCT vs. AIP as a reference IGRT.

*Patient Number, Fraction Numbers*	${\text{PTV}}_{100\%}$ *Using FBCT as a Reference IGRT*	${\text{PTV}}_{100\%}$ *Using AIP as a Reference IGRT*	*Difference (%)*
1, (1,3)	85.0	97.2	12.6
2, (3)	87.3	96.3	9.4
3, (2‐5)	93.9	97.4	3.5
4, (1‐5)	93.2	98.1	4.9
5, (1‐5)	91.9	96.0	4.3
6, (4‐5)	90.5	95.1	4.9

## IV. DISCUSSION

In this study, we investigated potential systematic uncertainties in IGRT by comparing the use of FBCT or AIP as the reference image set. After aligning to the bony anatomy, we found discrepancies in the COMs of the implanted fiducial markers between FBCT and AIP in the frame of weekly CBCT. Twenty fractions had COM discrepancies >3 mm in any direction. These discrepancies can be translated into a reduction in the PTV coverage.

The pancreatic tumor motion and displacement has been well quantified by many investigators.^4^, ^5^, ^6^, ^7^, ^8^ Our results from registering the implanted markers from the FBCT and AIP with the weekly CBCTs indicated that the interfractional tumor displacements were patient‐dependent and had large variations. Therefore, directly localizing the pancreatic tumor using the implanted markers on the AIP as a surrogate can reduce the required planning margins.

### A. FBCT vs. CBCT

FBCT is often used as the planning and reference CT for IGRT. After correcting setup errors with bony registration, the shifts in the implanted marker's COMs between FBCT and CBCT included the interfractional tumor displacement, the potential systematic uncertainties of the COMs of the markers, and the residual bony registration uncertainties. The range and spread of our data agreed with the published data.^4^, ^5^, ^6^, ^7^, ^8^

Whitfield et al.^(7)^ analyzed 109 kV CBCTs for 13 patients using surgical clips, six gold fiducial markers, and biliary stents, and analyzed the motion of a single point manually marked on the CBCT projections. They reported interfractional tumor displacements of 2.0 mm, 1.6 mm, and 2.6 mm in the RL, AP, and SI directions, respectively, which is 2–3 mm less than our data. Jayachandran et al.^(8)^ investigated the interfractional uncertainties for five patients with 140 fractions and five to six gold fiducial seeds using orthogonal kV images and fluoroscopy, and reported mean absolute shifts to fiducial markers after bony alignment of 1.8 mm, 1.6 mm, and 4.1 mm in the RL, AP, and SI directions, respectively, which is 1–2 mm less than our data.

Solla et al.^(6)^ studied the interfractional tumor displacement for 15 patients with single fiducial markers using weekly MV CBCTs (61 scans). The positions of fiducial markers after alignment to the bony anatomy were compared with the corresponding positions in the CBCT acquired in the first session. Their data range and root mean square for the tumor displacements (which they referred to as residual shifts) were comparable to our data in the RL and AP directions, but were within 2 mm in the SI direction.

These studies all used FBCT as the reference CT with variable sample sizes and techniques. Whitfield and colleagues used the standard deviation (SD) of the daily mean position of a single point (P), Jayachandran et al. reported results for five patients using orthogonal kV images, and Solla et al. used MV CBCT and a single fiducial marker. In our study, we analyzed 15 patients (a total of 75 kV CBCTs) with 3–5 implanted fiducial markers. All patients were treated with the same technique. Our results agreed with the published data in the range of pancreas motion from one fraction to another.

### B. AIP vs. CBCT

If AIP is used as the reference image for IGRT, after correcting for the setup errors with bony alignment, the shifts in the COMs of the implanted markers between AIP and CBCT included the interfractional tumor displacement and residual bony registration. Van der Horst et al.^(5)^ quantified the interfractional position variations using fiducial markers on daily kV CBCT for 13 patients (300 CBCTs). They manually registered the markers on CBCTs to the reference CT, which in their study was AIP for 10 patients and FBCT for three patients. In our study, we registered the CBCT with AIP to the bony structure and quantified the differences between the marker COMs on both data sets. The Van der Horst study reported group systematic errors of 3.9 mm, 3.6 mm, and 5.5 mm in RL, AP, and SI directions, respectively, which agreed with our data of 3.2 mm, 3.3 mm, and 6.3 mm in the RL, AP, and SI directions, respectively.

### C. COM differences in FBCT/CBCT and AIP/CBCT

The acquisition of CBCT takes about 1 to 2 min, which includes many respiratory cycles. The COMs of implanted markers represent an average respiratory tumor position, similar to the AIP from 4D CT. On the other hand, FBCTs are acquired in a short time, which can capture the implanted markers in any phase of the respiratory cycle. In this study, we quantified the COM discrepancies between FBCT and AIP in the reference frame of the same CBCT to estimate the systematic uncertainties that may result from using FBCT as the reference CT for image‐guided tumor localization. The discrepancies in marker positions varied among patients (Fig. 2(c)) with ranges from −2.5 mm to 2.8 mm, −2.9 mm to 3.7 mm, and −6.0 mm to 10.5 mm in the RL, AP, and SI directions, respectively.

The greatest pancreatic motion is in the SI direction, and discrepancies in the COMs of the implanted markers when using FBCT or AIP as reference images for IGRT can impact the dosimetry of the PTV and the surrounding critical organs. For the fractions with COM discrepancies of the implanted markers >3 mm in any directions, the dose coverage to the PTV could be reduced significantly. The PTV was constructed to account for the setup and tumor motion uncertainties. In this study, we showed a potential systematic uncertainty that may not be accounted for in the PTV. For the surrounding normal tissues, no clinical differences were noticed for these fractions because a large planning margin was used in the treatment planning.

In clinical practice, one can directly use AIP as the planning and reference CT for IGRT. This may introduce some clinical challenges, including degraded image quality and potential inaccuracy of electronic density for planning. Han et al.^(9)^ compared AIP with FBCT for contouring organs at risk and radiation treatment planning for stereotactic‐body radiation therapy (SBRT) in the lung and found no significant differences between the two sets. Tian et al.^(10)^ compared AIP and FBCT for treatment planning and dose calculations for lung SBRT and concluded that the dosimetric characteristics are similar for both datasets. Similar studies for pancreatic tumors are needed to demonstrate the feasibility of using AIP for treatment planning and IGRT. Another challenge is that, because AIP is a synthetic CT, some CT simulators may not be able to create AIP and support the function of direct placing of the isocenter on the synthetic CT during virtual simulation.

## V. CONCLUSIONS

In this study, we analyzed the systematic uncertainties that resulted from using FBCT as a reference image set for IGRT of pancreatic tumor patients. CBCT represents the average breathing motion of the patient, and the use of AIP as a reference image set is recommended to reduce the uncertainties observed in FBCT. If FBCT is used as a reference image, additional uncertainties need to be considered.

## ACKNOWLEDGMENTS

This research was supported in part by a subaward from The Johns Hopkins University with funds provided by the National Cancer Institute. It is also supported in part by Siemens Medical Solutions.

## Supporting information

Supplementary Material
